# Vinegar‐Processed Black Soybean Promotes Hair Growth and Prevents Alopecia via Wnt/β‐Catenin Pathway

**DOI:** 10.1002/fsn3.70625

**Published:** 2025-07-16

**Authors:** Yue Zhang, Miaomiao Wu, Jiasen Li, Chenghao Zhao, Tingyuan Zhou, Huiling Wei, Jiaxin He, Yao Yao, Juan Li

**Affiliations:** ^1^ School of Pharmacy Ningxia Medical University Yinchuan People's Republic of China; ^2^ School of Basic Medical Sciences Ningxia Medical University Yinchuan People's Republic of China; ^3^ Ningxia Engineering and Technology Research Center for Modernization of Characteristic Chinese Medicine, and Key Laboratory of Ningxia Ethnomedicine Modernization, Ministry of Education Ningxia Medical University Yinchuan People's Republic of China

**Keywords:** antialopecia, functional food, hair regeneration, vinegar‐processed black soybean, Wnt/β‐catenin signaling pathway

## Abstract

The escalating prevalence of alopecia, exacerbated by rising life stressors, significantly impacts individuals' physical appearance and psychological well‐being, highlighting the need for effective interventions. Black soybean ( *Glycine max* [L.] Merr.) is widely used in medicinal and dietary fields with a high safety profile. This study investigated the effects and mechanisms of vinegar‐processed black soybean (VPBS) and its aqueous extract (VPBS‐AE) on hair regeneration and antialopecia in C57BL/6J mice. For hair regeneration assessment, sodium sulfide‐depilated mice were orally administered VPBS (0.75, 1.5, or 3 g·kg^−1^·day^−1^) or VPBS‐AE (0.075, 0.15, or 0.3 g·kg^−1^·day^−1^) for 18 days. Outcomes were evaluated via trichogram scoring, hair length and weight analysis, histomorphometric evaluation, and Western blot quantification of β‐catenin, GSK3β, and Wnt10b expression. To assess antialopecia effects, cyclophosphamide (100 mg·kg^−1^) was intraperitoneally administered on Day 9 postdepilation, followed by 6‐day oral administration of VPBS (1.5, 3, or 6 g·kg^−1^·day^−1^) or VPBS‐AE (0.15, 0.3, or 0.6 g·kg^−1^·day^−1^). Alopecia severity was determined through trichogram analysis and macroscopic scoring. Results demonstrated that VPBS dose‐dependently accelerated hair growth, stimulated anagen phase entry, and attenuated cyclophosphamide‐induced alopecia. VPBS‐AE similarly promoted follicular repair, delayed alopecia, and preserved follicle integrity. Western blotting analysis demonstrated that in the model group, sodium hydrosulfide‐induced alopecia led to significant downregulation of Wnt10b and β‐catenin, and upregulation of GSK‐3β. While VPBS‐AE treatment effectively restored the expression levels of Wnt10b/β‐catenin and suppressed GSK‐3β expression, suggesting the therapeutic effects of VPBS‐AE may be mediated through the Wnt/β‐catenin pathway. Our findings substantiate VPBS and VPBS‐AE as promising functional food candidates for hair regeneration and alopecia prevention, with mechanistic insights implicating Wnt/β‐catenin signaling modulation.

## Graphical abstract

Vinegar‐processed black soybean (VPBS) and its aqueous extract (VPBS‐AE) dose‐dependently accelerated hair growth, stimulated anagen phase entry, and attenuated cyclophosphamide‐induced alopecia. VPBS‐AE promoted follicular repair, delayed alopecia, and preserved follicle integrity. These effects may be mediated through activation of the Wnt/β‐catenin pathway.

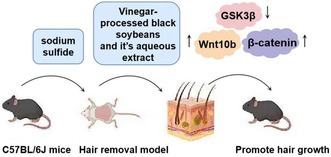

## 1 Introduction

Alopecia is a prevalent and challenging condition in dermatology. Although not life‐threatening, it profoundly impacts individuals' psychosocial well‐being and self‐perception. Research indicates that up to 62% of patients experience various disruptions in their lives due to alopecia, including impacts on interpersonal relationships, educational attainment, and career opportunities. Affected individuals frequently encounter social stigma, which exacerbates psychological distress and diminishes overall quality of life (Schielein et al. 2020). Furthermore, pediatric and adolescent populations with alopecia are disproportionately vulnerable to bullying, ranging from verbal harassment to physical aggression (Rudnicka 2023). These factors collectively underscore the urgent need for research into effective interventions for alopecia.

Hair originates from the ectodermal layer of the skin and serves as an important component of the body (Truong et al. 2021). Mammalian hair growth progresses cyclically through three phases: anagen (growth phase), catagen (regression phase), and telogen (resting phase) (Chai et al. 2019; Ahmed et al. 2017; Harshuk‐Shabso et al. 2020). During anagen, follicular keratinocytes actively proliferate, driving robust hair shaft elongation. Catagen involves follicular involution, while telogen represents a quiescent transitional phase preceding renewed anagen initiation (Jeong et al. 2022). Alopecia is commonly categorized as primary (follicle‐centric, idiopathic) or secondary (associated with systemic disease, pharmacotherapy, or trauma). Although its pathogenesis remains incompletely elucidated, key contributors include hormonal dysregulation, autoimmune responses, follicular cycle disruption, and structural damage to hair‐forming units. The dermal papilla, a mesenchymal niche within the follicle, orchestrates follicular cycling via paracrine signaling, secreting cytokines and morphogens that regulate proliferation and differentiation. Notably, the Wnt/β‐catenin pathway has been identified as a pivotal regulator of dermal papilla cell activation and follicular regeneration, establishing it as one of the key signaling pathways in hair growth modulation (Fresta et al. 2020).

Currently, alopecia treatment primarily consists of two categories: nonpharmacological and pharmacological therapies (Ocampo‐Garza et al. 2019). For nonpharmacological treatments, methods such as hair transplantation are predominantly employed (Jimenez et al. 2021). Pharmacological treatments target follicular atrophy or growth inhibition by modulating key signaling pathways. Clinically utilized modalities include scalp micropigmentation, platelet‐rich plasma injections, and topical corticosteroids, with adjunctive therapies such as minoxidil and finasteride demonstrating efficacy (Salim and Kamalasanan 2020). While oral finasteride for androgenetic alopecia is approved by the Drug Administration (FDA) and the European Medicines Agency (EMA), safety concerns have shifted research focus toward topical formulations (Kim et al. 2019). Minoxidil, the sole FDA‐approved over‐the‐counter treatment, is associated with adverse effects including hypertrichosis, scalp irritation, erythema, and desquamation (Badria et al. 2020; Fresta et al. 2020). Emerging therapies such as JAK inhibitors and PGF2 analogs show promise but face challenges in therapeutic efficacy, safety profiles, and long‐term outcomes, necessitating further investigation (Wang et al. 2018; Chen et al. 2021; Ocampo‐Garza et al. 2019; Iorizzo and Tosti 2018). Recently, botanical extracts including nettle, saw palmetto, grape seed, and pumpkin seed exhibit antialopecia potential, underscoring the therapeutic value of phytochemicals in alopecia management (Gasmi et al. 2023; Kim et al. 2024). Preclinical studies highlight natural compounds as viable candidates for promoting hair regeneration and mitigating follicular damage (Leem et al. 2018).

Black soybean ( *Glycine max* [L.] Merr.) produces nutrient‐dense seeds renowned for their medicinal and dietary applications with a high safety profile. Black soybean is a rich source of energy, protein (containing up to 49.8%—the highest among legumes), dietary fiber, essential amino acids, vitamins, and minerals (Nguyen 2023). Pharmacologically, black soybean exhibits antioxidative, anti‐inflammatory, antiaging, and metabolic regulatory properties, as well as antiproliferative activity against cancer cells and cardioprotective benefits (Fonseca Hernández et al. 2023; Cheng et al. 2024; Liu et al. 2022; Li et al. 2023; Koh et al. 2023; Zheng et al. 2023). Notably, black soybean shows the ability to inhibit premature hair graying, while its nontoxic and eco‐friendly nature positions black soybean‐based hair dyes as sustainable alternatives to conventional products (Inman et al. 2020). Despite these attributes, research on their hair growth‐promoting potential remains limited. In vitro studies suggest that black soybean extracts and fermented derivatives enhance hair follicle dermal papilla cell (HFDPc) proliferation by activating hair growth‐related signaling pathways (Choi et al. 2017). Preliminary clinical evidence further supports their efficacy in stimulating hair regeneration and mitigating alopecia (Sung and Kim 2022).

Vinegar processing is a technique integral to traditional Chinese medicine (TCM) preparation (Gao et al. 2020; Yatagai et al. 2002). In the theory of TCM processing, vinegar processing represents a significant technique for treating TCM materials within an acidic environment. According to TCM principles, vinegar processing is believed to target the liver meridian. The liver, in TCM theory, plays a crucial role in blood storage, and its condition is reflected in the health of the hair. Vinegar processing can enhance the specificity of drugs for the liver meridian, thereby indirectly alleviating hair loss caused by blood deficiency (Zhang et al. 2022). In addition, as a polar solvent, acetic acid can disrupt the structure of plant cell walls, facilitating the dissolution of water‐soluble components, including phenolic compounds and flavonoid glycosides. This process increases the bioavailability of active ingredients (Islas et al. 2012; Chen et al. 2023). These bioactive components possess antioxidant and anti‐inflammatory properties, which contribute to the improvement of the hair follicle microenvironment (Park et al. 2022). Despite these established effects of vinegar processing, the effects of vinegar‐processed black soybean (VPBS) on hair regeneration and alopecia prevention remain uninvestigated.

This study was designed to evaluate the effect of VPBS and its aqueous extract (VPBS‐AE) on promoting hair growth and preventing alopecia, with a subsequent exploration of the underlying mechanisms. In the study, hair growth and alopecia mouse models were orally administered different doses of VPBS and VPBS‐AE. Through multiple methods including hair growth scoring, quantitative analysis of hair length and weight, observation of skin tissue morphology, and detection of the protein expression levels of β‐catenin, glycogen synthase kinase 3β (GSK‐3β) and Wnt10b via Western blotting analysis, the hair‐regenerative and antialopecia properties of VPBS and VPBS‐AE were explored for the first time.

## 2 Materials and Methods

### 2.1 Preparation of VPBS and VPBS‐AE

#### 2.1.1 Preparation of VPBS

Black soybeans were rinsed repeatedly with distilled water until foam ceased, followed by thorough drainage and air‐drying at ambient temperature. The cleaned soybeans were steamed for 1 h, after which they were dehydrated at 45°C for 2 h. The dried soybeans were subsequently immersed in 9° white vinegar at a 1.5:1 (v/w) vinegar‐to‐bean ratio and macerated under light‐protected conditions at 25°C for 14 days. Postmaceration, the beans were transferred to sterile trays and desiccated in a forced‐air drying oven at 45°C for 48 h. The final product was pulverized using a high‐speed herbal grinder and sieved through a 100‐mesh screen. The resulting VPBS powder was stored in airtight containers at 4°C until further use.

#### 2.1.2 Preparation of VPBS‐AE and Residue

The pulverized VPBS powder was extracted with distilled water (1:10 w/v) using ultrasonic‐assisted extraction (40 kHz, 300 W) for 30 min, followed by vacuum filtration to isolate the supernatant and solid residue. The extraction cycle was repeated three times to maximize yield. Pooled filtrates were concentrated via rotary evaporation and lyophilized to obtain VPBS‐AE powder. The solid residue was lyophilized to obtain VPBS‐AE residue. The extraction yield of VPBS‐AE is 10%, and the residue yield is 90% (1 g VPBS‐AE and 9 g residue can be obtained from 10 g VPBS). The VPBS‐AE and residue were stored in airtight containers at 4°C until experimental use.

### 2.2 Animal Experiments

#### 2.2.1 Animals

SPF grade female C57BL/6J mice (Chen et al. 2016; Lim et al. 2019), body weight 16–18 g, were obtained from the Laboratory Animal Center of Ningxia Medical University. All animal procedures were performed in accordance with the Provision and General Recommendation of Chinese Experimental Animals Administration Legislation and approved by the Ethics Committee of Ningxia Medical University (Approval No. 2022‐Z030). Mice were housed under SPF conditions in a temperature‐ and humidity‐controlled environment (22°C ± 1°C, 45%–55% relative humidity) with a standardized 12‐h light/dark cycle. Food and water were provided ad libitum throughout the study. Androgenetic alopecia, a common hair loss disorder, is primarily caused by male hormones (Kim et al. 2023). Given that male mice generally have higher androgen levels than female mice, to minimize the potential confounding influence of androgens on the experimental results, female mice were specifically chosen as the experimental model in this study.

#### 2.2.2 Experimental Procedure for Hair Growth Promotion

A total of 48 healthy female C57BL/6J mice were randomly divided into six groups: control group, model group, low, medium, high‐dose groups of VPBS (0.75, 1.5, and 3 g/kg, respectively), and alopecia areata pill (Ban Tu Wan, BTW, positive control, Guangzhou Baiyunshan Jingxiutang Pharmaceutical Co. Ltd., Lot No. R07019) group (3.8 g/kg), with eight mice in each group. The positive control BTW is frequently used in clinical treatment of alopecia areata, demonstrating remarkable therapeutic efficacy (Lu and Zhou 2007; Wang and Wang 2016). Alopecia was induced in all groups except the control by topical application of 10% sodium sulfide solution (He et al. 2022). Briefly, mice were anesthetized via intraperitoneal injection of pentobarbital sodium (50 mg/kg). A 2 × 4 cm depilation area was delineated on the dorsal midline, shaved with a hair shaver, and treated with 10% sodium sulfide solution for 20 s. Residual depilatory agent was removed using sterile cotton swabs, followed by saline rinsing, drying, and placement under a heat lamp to prevent hypothermia (He et al. 2022; Zhou et al. 2022). The day of alopecia induction was designated as Day 0. From Day 1 onwards, the control and model groups received distilled water (20 mL/kg) via gavage, while the low, medium, and high‐dose groups of VPBS were administered 0.75, 1.5, and 3 g/kg VPBS, respectively. The positive control group received 3.8 g/kg of the BTW. Treatments were administered daily for 18 consecutive days. During the treatment period, photographs were taken, and hair regrowth was evaluated via macroscopic scoring at 48‐h intervals. On the 19th day, hair length and weight of the mice were measured (Begum et al. 2023; Deng et al. 2021; Zhou et al. 2022).

For the VPBS‐AE experiment, 48 female C57BL/6J mice were randomly divided into six groups: control group, model group, VPBS group (1.5 g/kg), VPBS‐AE group (0.15 g/kg), VPBS‐AE residue group (1.35 g/kg), and BTW group (3.8 g/kg, positive control), with eight mice in each group. The remaining experimental procedures were consistent with those applied to the VPBS. Subsequently, we conducted a study to evaluate the effects of various doses of the aqueous extract, utilizing the same number of mice in each experiment, which were subsequently divided into low, medium, and high dosage groups (0.075, 0.15, and 0.3 g/kg). The extraction yield of the VPBS‐AE was approximately 10%. On the 19th day, skin samples were collected from the experimental area of the mice for hematoxylin and eosin (H&E) staining and Western blot assays. The remaining experimental procedures were consistent with those applied to the VPBS.

#### 2.2.3 Experimental Procedure for Anti‐Alopecia Experiments

A total of 48 female C57BL/6J mice were randomly divided into six groups: control group, model group, three dosage groups of VPBS (1.5, 3, and 6 g/kg), and BTW group (3.8 g/kg, positive control), comprising eight mice per group. Depilation was induced in all groups with topical 10% sodium sulfide application, as described in Section 2.2.2. The day of alopecia induction was designated as Day 0. On Day 9, all groups except the control group received an intraperitoneal injection of cyclophosphamide (100 mg/kg) to model chemotherapy‐induced alopecia (Yoneda et al. 2021; Paus et al. 1994, 1996; Plonka et al. 2005; Chen et al. 2016; Lim et al. 2019). From Day 10 onwards, the control and model groups were administered distilled water (20 mL/kg) via gavage, while the low, medium, and high dosage VPBS groups were gavaged with 1.5, 3, and 6 g/kg VPBS, respectively. The positive control group was gavaged with 3.8 g/kg of BTW. The intervention lasted 6 consecutive days. Alopecia progression was documented macroscopically at 48‐h intervals through standardized photographic imaging.

The VPBS‐AE was administered to equal numbers of mice divided into low, medium, and high‐dose groups (0.15, 0.3, and 0.6 g/kg). The experimental methods were consistent with those used for VPBS.

### 2.3 Hair Growth Status Analysis

#### 2.3.1 Measurement of Hair Length

Hair length within the depilated area was measured using a vernier caliper. Three distinct regions in the experimental region were selected, and the 10 randomly selected hairs from each region were measured for statistical analysis (Begum et al. 2023; Deng et al. 2021; Zhou et al. 2022).

#### 2.3.2 Hair Weight Quantification

All hairs in the depilated area were carefully shaved, collected onto adhesive‐coated, preweighed tape, and transferred to an analytical balance to determine net weight (Begum et al. 2023; Deng et al. 2021; Zhou et al. 2022).

#### 2.3.3 Hair Growth Scoring Criteria

Hair regeneration was scored on a validated 0–3 scale: a score of 0 denotes no hair growth; a score of 1 indicates the presence of thin hair in the depilated area; a score of 2 is assigned when the length and density of hair in the depilated area are approximately half that of the nondepilated area; a score of 3 is given when the depilated area exhibits hair comparable to that of the nondepilated area. Mice displaying irregular hair growth are scored based on the area ratio (Miao et al. 2020; Wang et al. 2024; Zhou et al. 2022).

#### 2.3.4 Alopecia Scoring Criteria

For alopecia evaluation, the following scoring criteria (Bechard et al. 2011) were adopted: a score of 0 was assigned for no hair loss; score 1 was given for hair loss on the head and neck; score 2 was assigned when there was sparse hair loss on the head, neck, and back; score 3 was allotted for significant hair loss on the head, neck, and back; and score 4 was designated for complete hair loss on the head, neck, and back.

### 2.4 H&E Staining

On Day 19, mice were euthanized via intraperitoneal injection of pentobarbital sodium (150 mg/kg). Dorsal skin tissues encompassing the depilated area were excised, rinsed in phosphate‐buffered saline (PBS), and fixed in 4% paraformaldehyde (PFA) for 12 h at 4°C, followed by an additional 12 h fixation in fresh PFA. Tissues were trimmed into 1 × 1 cm^2^ sections, bisected sagittally to yield transverse and longitudinal blocks, and dehydrated through a graded ethanol series. Samples were paraffin‐embedded, and 4‐μm‐thick sections were cut using a rotary microtome. Sections were mounted on glass slides, dried at 60°C for 2 h, and deparaffinized in xylene. The sections were then stained with H&E, sealed with neutral resin, and observed under a light microscope.

### 2.5 Western Blot Analysis

Total protein was extracted from murine dorsal skin tissues using a Total Protein Extraction Kit (Servicebio, China), following the manufacturer's instructions. Protein concentration was quantified via the bicinchoninic acid (BCA) assay (Servicebio), ChinaEqual amounts of protein were resolved by 10% or 12% SDS‐PAGE and electrophoretically transferred to PVDF membranes. Membranes were blocked with 5% nonfat milk in Tris‐buffered saline containing 0.1% Tween‐20 (TBST) for 2 h at room temperature. The membranes were then incubated overnight at 4°C with primary antibodies against Wnt10b (1:2000, Affinity, DF9038), GSK3β (1:1500, Affinity, AF2016), β‐catenin (1:1000, Santa Cruz, sc‐53,483) and β‐actin (1:5000, Affinity, AF7018). After three TBST washes, membranes were incubated with horseradish peroxidase (HRP)‐conjugated antirabbit IgG secondary antibodies (1:50000, Abbkine, A21020, or 1:6000, Affinity, S0002) for 1 h at room temperature. Following three additional TBST washes, immunoreactive bands were visualized using the New‐SuperECL detection kit (KeyGEN, Nanjing, China) and imaged with a SH‐Focus523 chemiluminescence system (Hangzhou Shen Hua Technology). Band intensity was quantified using ImageJ software (version 1.54 m, NIH, MD, USA).

### 2.6 Statistical Analysis

GraphPad Prism (version 8.3.0, GraphPad software, CA, USA) was used for statistical analysis. Data were obtained from multiple repeats of different biological experiments to obtain the mean values and standard error of the mean (SEM) displayed throughout. After normality confirmation, comparisons between multiple groups were made by one‐way ANOVA followed by Tukey's post hoc test. A value of *p* < 0.05 was considered statistically significant.

## 3 Results

### 3.1 Effects of VPBS on Hair Growth in C57BL/6J Mice

**FIGURE 1:**
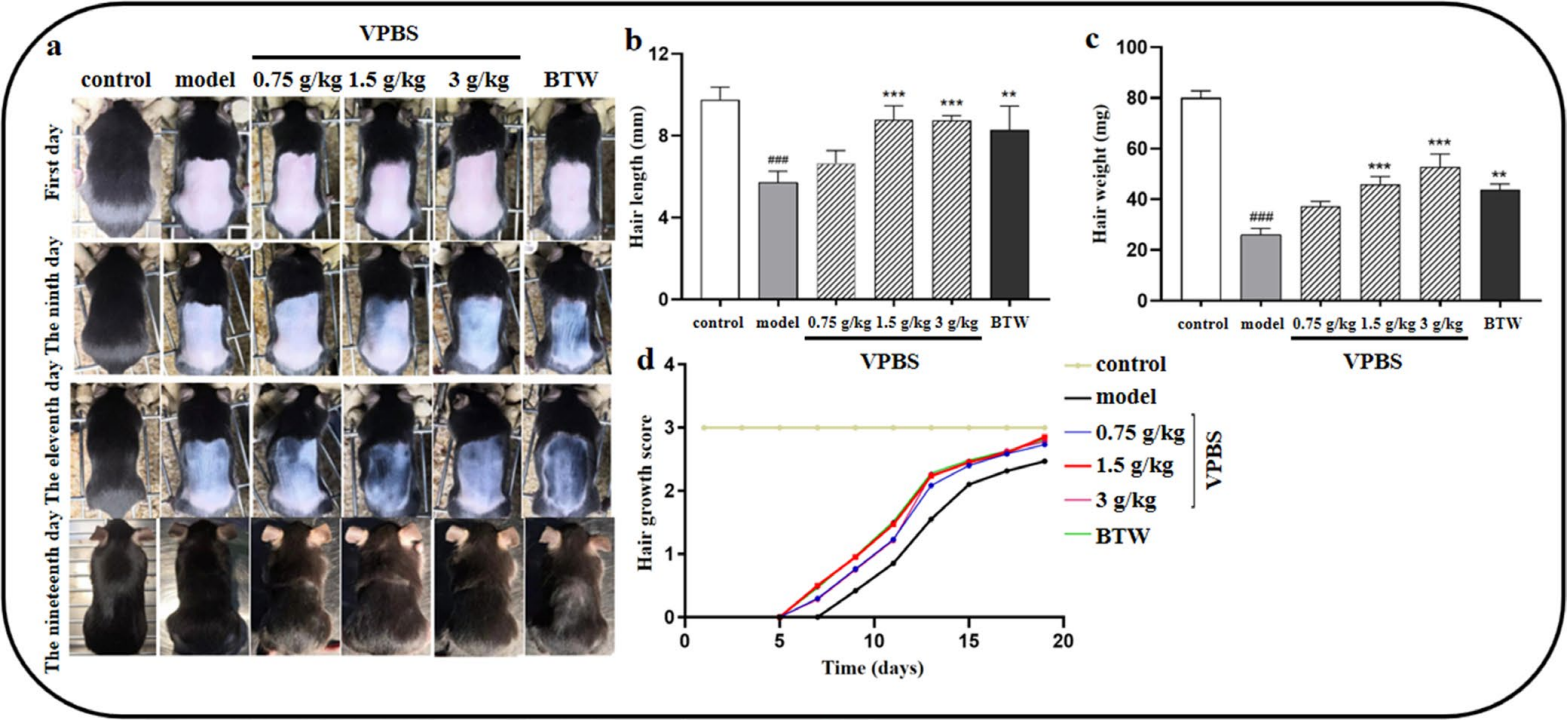
Effects of different doses of VPBS on hair growth of C57BL/6J mice. (a) Hair growth in C57BL/6J mice at different days. (b) Effect of VPBS on hair length of C57BL/6J mice. (c) Effect of VPBS on hair weight of C57BL/6J mice. Compared with the control group, ^###^ *p* < 0.001; compared with the model group, **p < 0.01, ***p < 0.001. (d) Effect of VPBS on hair growth score of C57BL/6J mice. The scoring criteria (values accurate to 1 decimal place) is as following: Score of 0 denotes no hair growth; score 1 indicates the presence of thin hair in depilated area; score 2 is assigned when the length and density of hair in the depilated area are approximately half that of the nondepilated area; score 3 is given when the depilated area exhibits hair comparable to that of the nondepilated area.

On Day 0, depilation with sodium sulfide solution induced complete hair removal in all groups except the control. VPBS‐treated groups (1.5 and 3 g/kg) exhibited promoted hair growth compared to the model group on Days 9, 11, and 19 (Figure 1a). Compared to the model group, mice in the 1.5 and 3 g/kg VPBS treatment groups exhibited a significant increase in hair length and hair weight, while no significant difference was observed in the 0.75 g/kg group (Figure 1b,c). Furthermore, 1.5 and 3 g/kg VPBS treatment mice showed higher hair growth scores compared to the model group (Figure 1d).

### 3.2 Effects of VPBS on Alopecia

**FIGURE 2:**
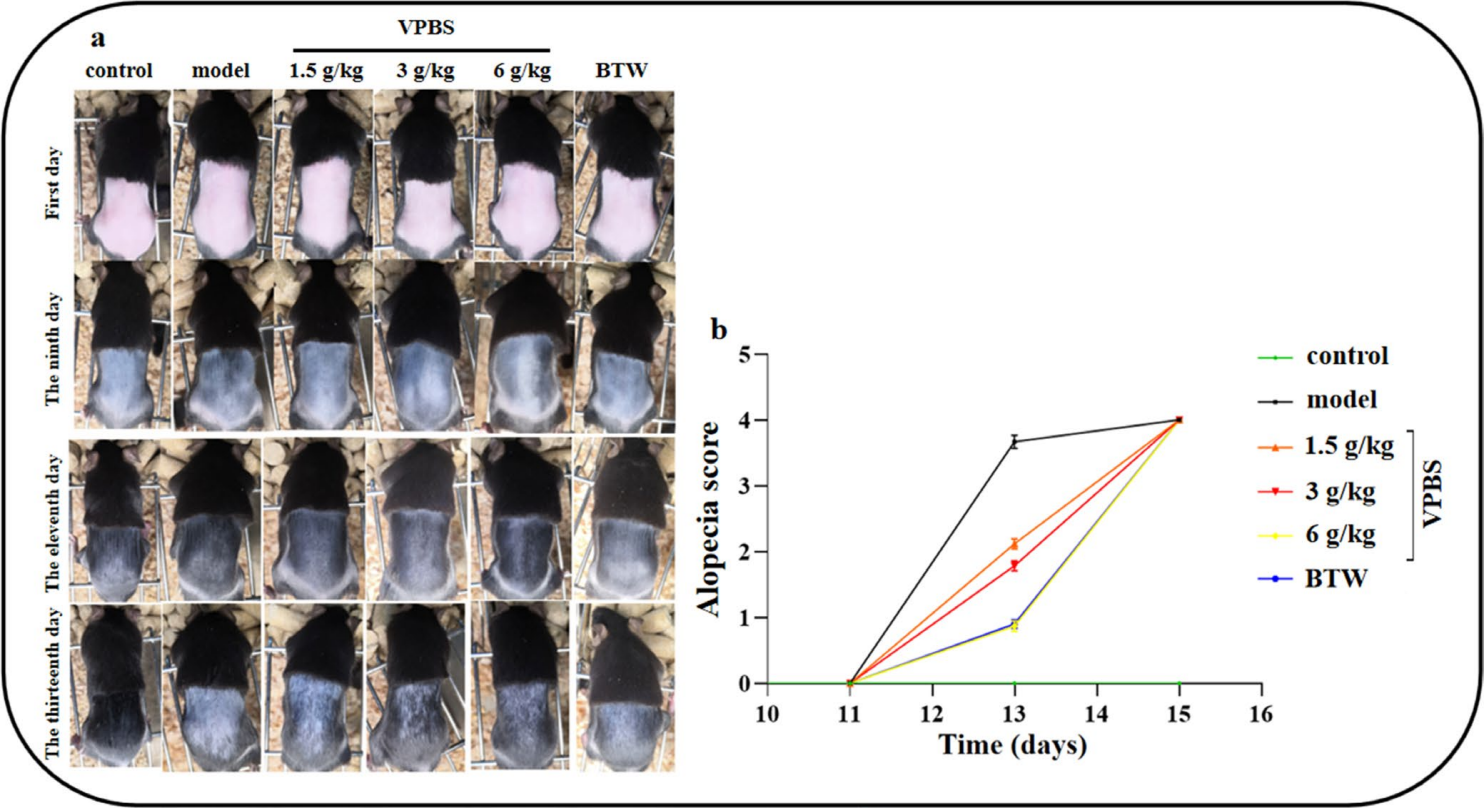
Effects of different doses of VPBS on cyclophosphamide‐induced alopecia in C57BL/6J mice. (a) Alopecia in C57BL/6J mice at different days. (b) Effect of VPBS on alopecia score of C57BL/6J mice. Scoring criteria: A score of 0 was assigned for no hair loss; score 1 was given for hair loss on the head and neck; score 2 was assigned when there was sparse hair loss on the head, neck, and back; score 3 was allotted for significant hair loss on the head, neck, and back; and score 4 was designated for complete hair loss on the head, neck, and back.

On Day 9, cyclophosphamide was injected intraperitoneally to establish a chemotherapy‐induced alopecia model. By Day 12, a substantial area of alopecia was observed in the model group, which was ameliorated following the administration of VPBS (Figure 2a). Meanwhile, the alopecia scores in VPBS‐treated groups significantly decreased from Days 12–13, compared to the model group (Figure 2b). These results demonstrate that VPBS can inhibit cyclophosphamide‐induced alopecia.

### 3.3 Effect of Different VPBS Extracts on Hair Growth

**FIGURE 3:**
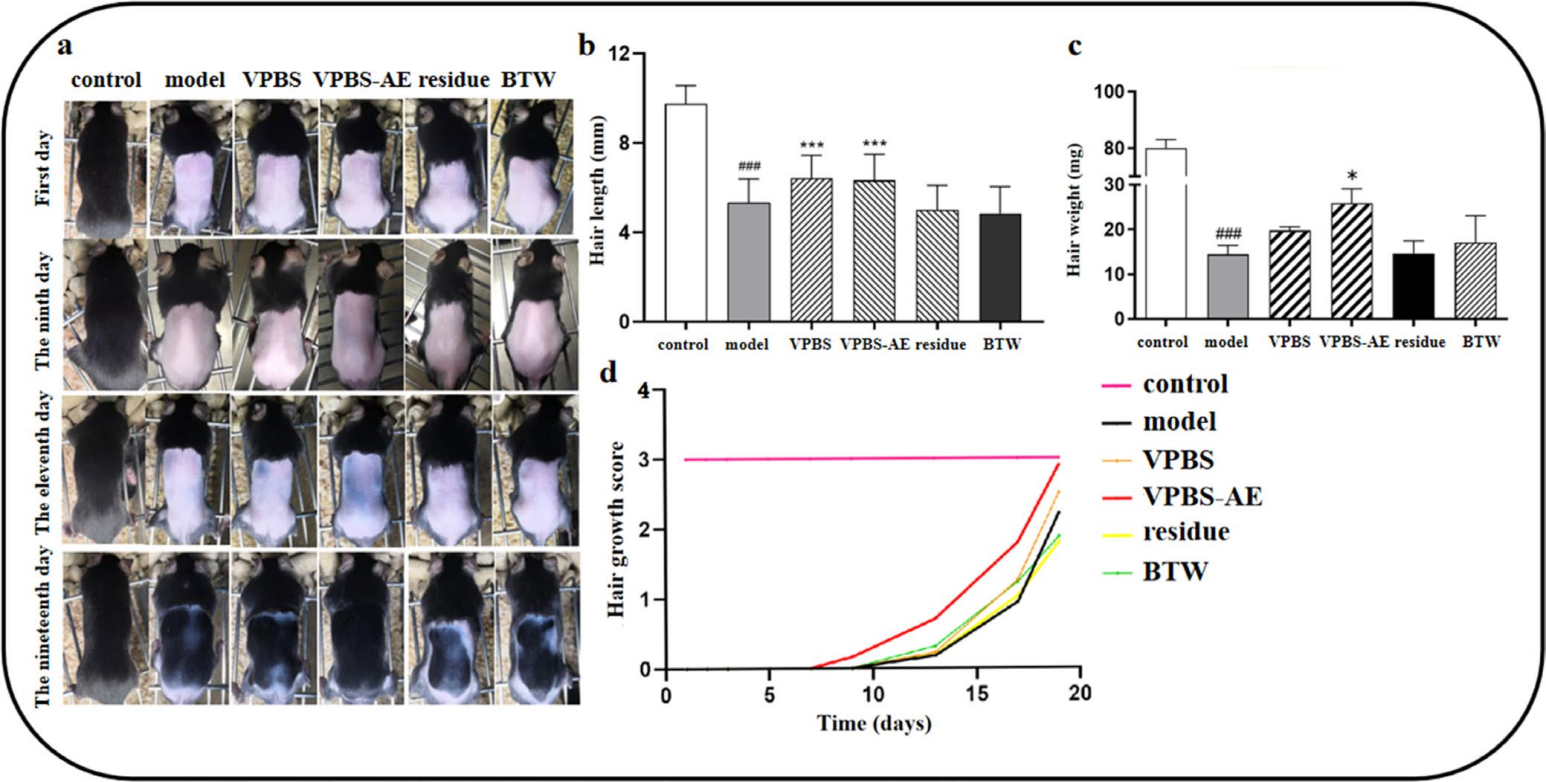
Effects of VPBS and its different extractions on hair growth of C57BL/6J mice: (a) Hair growth in C57BL/6J mice at 19 days; (b) effect of VPBS and its different extractions on hair length of C57BL/6J mice; (c) effect of VPBS and its different extractions on hair weight of C57BL/6J mice; (d) effect of VPBS and its different extractions on hair growth score of C57BL/6J mice. Compared with control group, ^###^ *p* < 0.001; compared with model group, *p < 0.05, ***p < 0.001.

VPBS‐AE treatment exhibited promoted hair growth, increased hair length, and hair weight on Days 9, 11, and 19, compared to the model group (Figure 3a–c). In contrast, no significant difference was observed in the VPBS residue group (Figure 3b). Additionally, the VPBS‐AE mice showed a higher hair growth score compared to the model group (Figure 3d). These results suggest that VPBS‐AE is the active fraction in VPBS that promotes hair growth.

### 3.4 Effects of VPBS‐AE on Hair Growth

**FIGURE 4:**
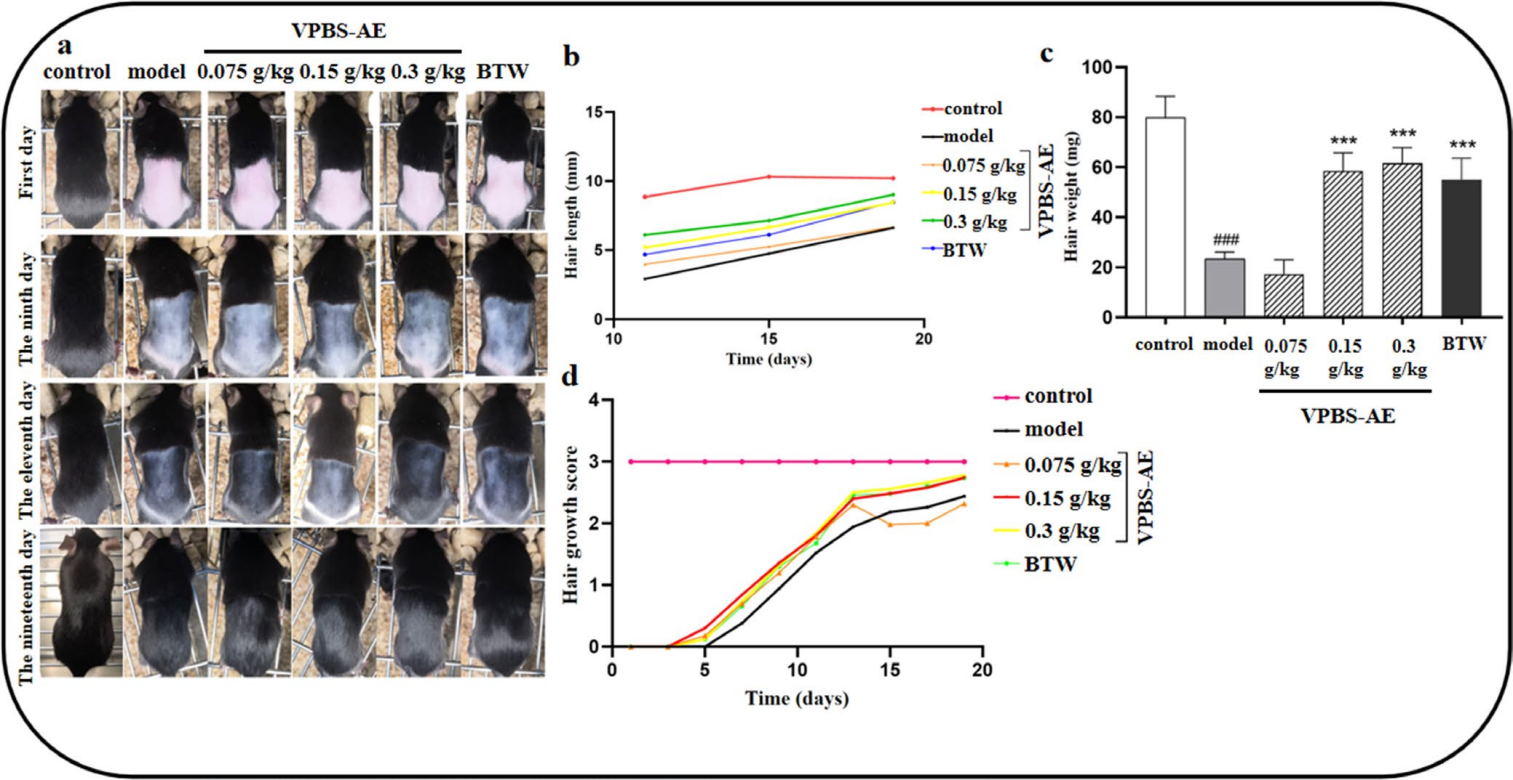
Effects of different doses of VPBS‐AE on hair growth of C57BL/6J mice. (a) Hair growth in C57BL/6J mice at 19 days. (b) Effects of different doses of VPBS‐AE on hair length of C57BL/6J mice. (c) Effects of different doses of VPBS‐AE on hair weight of C57BL/6J mice. (d) Effects of different doses of VPBS‐AE on hair growth score of C57BL/6J mice. Compared with control group, ^###^ *p* < 0.001; compared with model group, ***p < 0.001.

VPBS‐AE treatment (0.15 and 0.3 g/kg) exhibited promoted hair growth, increased hair length, and hair weight on Days 9, 11, and 19, compared to the model group (Figure 4a–c). Additionally, the VPBS‐AE mice showed a higher hair growth score compared to the model group (Figure 4d).

**FIGURE 5:**
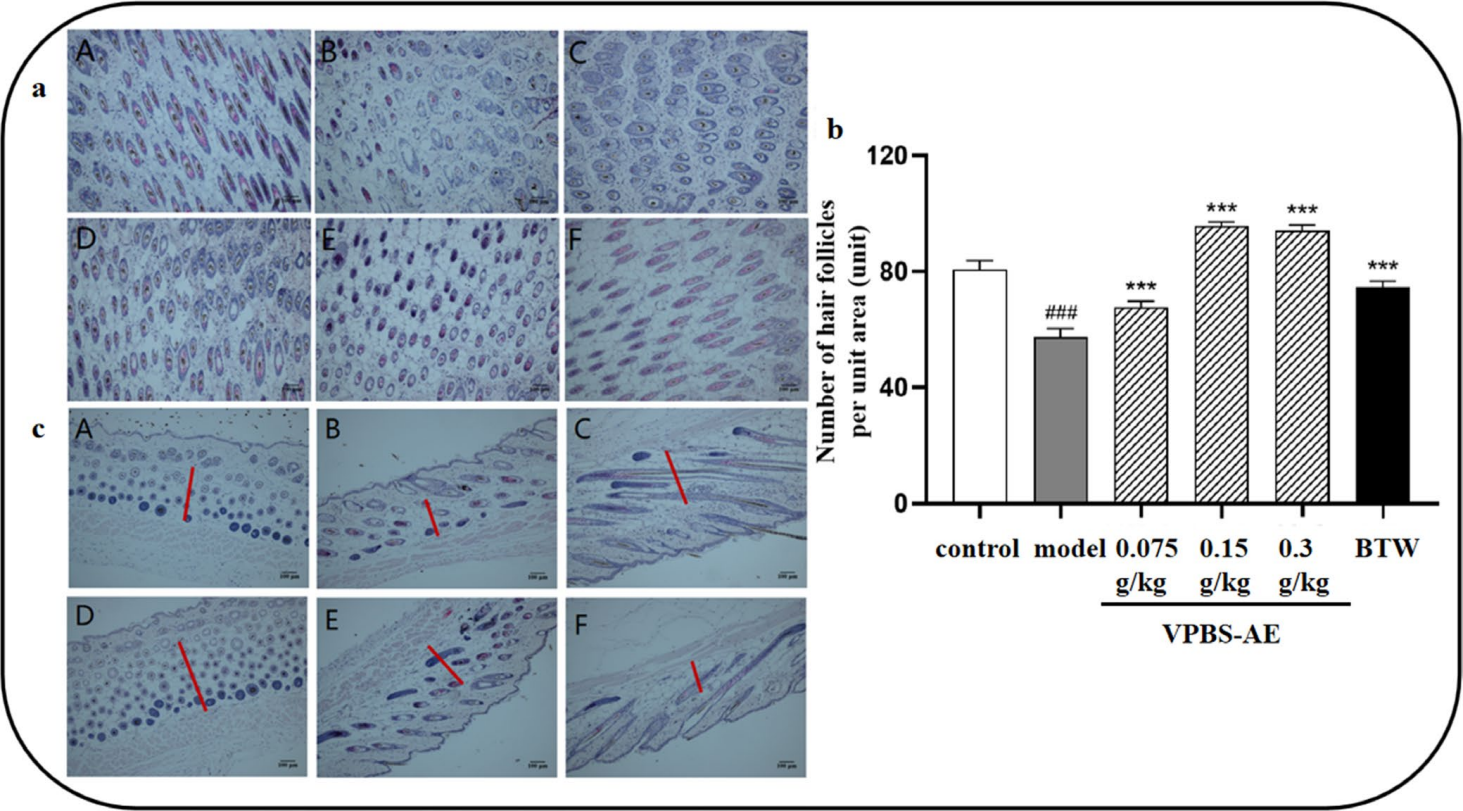
H&E staining of dorsal skin tissues in depilated area of mice. (a) A longitudinal section of H&E‐stained skin tissue taken from the back of mice. (b) The number of hair follicles per unit area of skin tissue in the experimental area of mice for 19 days after administration of VPBS‐AE (compared with control group, ^###^ *p* < 0.001, compared with model group, ***p < 0.001). (c) A transverse section of H&E‐stained skin tissue derived from the dorsal area of mice. The length of the red line indicates the thickness of the dermis. H&E staining of back skin tissue of mice: (A) control group; (B) model group; (C) VPBS‐AE 0.75 g/kg; (D) VPBS‐AE 1.5 g/kg; (E) VPBS‐AE 3 g/kg; (F) BTW, 3.8 g/kg (magnification ×100, scale bar = 100 μm).

Histopathological analysis via H&E staining revealed sparse hair follicle distribution in model group mice, accompanied by follicular atrophy and vacuolation. VPBS‐AE administration substantially improved follicular architecture, increasing follicular density while reducing vacuolation (Figure 5a). Quantitative analysis demonstrated that both 0.15 and 0.3 g/kg VPBS‐AE significantly enhanced hair follicle count per unit area compared to the model group, surpassing the efficacy observed in the positive control group (Figure 5b). Furthermore, dermal thickness was markedly increased in VPBS‐AE treatment groups compared to model mice (Figure 5c).

### 3.5 Effects of VPBS‐AE on Expression of Proteins in Wnt/β‐Catenin Pathway

**FIGURE 6:**
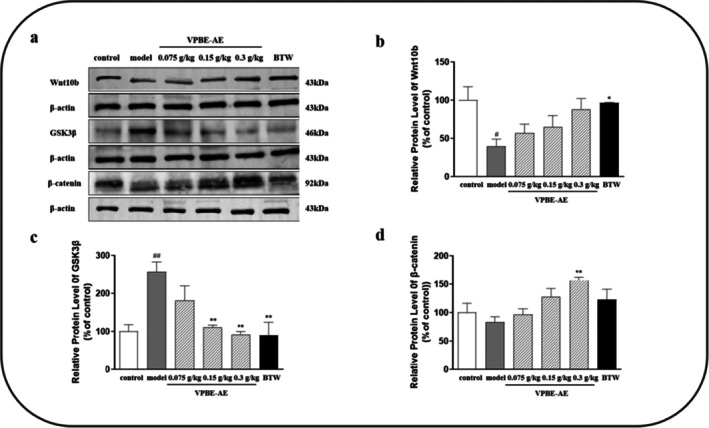
The effect of VPBS‐AE on the expression of hair growth‐related proteins in the skin tissues of C57BL/6J mice (*n* = 3). (a) Effects of VPBS‐AE on the expression of Wnt10b, GSK3β, and β‐catenin; (b) quantitative analysis of the relative expression levels of Went10b; (c) quantitative analysis of the relative expression levels of GSK3β; (d) quantitative analysis of the relative expression levels of β‐catenin protein. Compared with the control group, ^#^ *p* < 0.05, ^##^ *p* < 0.01; compared with the model group, *p < 0.05, **p < 0.01.

Western blot analysis revealed that sodium hydrosulfide application in the model group significantly downregulated Wnt10b and β‐catenin protein expression, while upregulating GSK3β levels compared to the control group (Figure 6). In contrast, VPBS‐AE treatment reversed these effects, restoring Wnt10b and β‐catenin expression and suppressing GSK3β levels in dorsal skin tissues. These findings suggest that VPBS‐AE may promote hair growth via the Wnt/β‐catenin pathway.

### 3.6 Effect of VPBS‐AE on Cyclophosphamide‐Induced Alopecia

**FIGURE 7:**
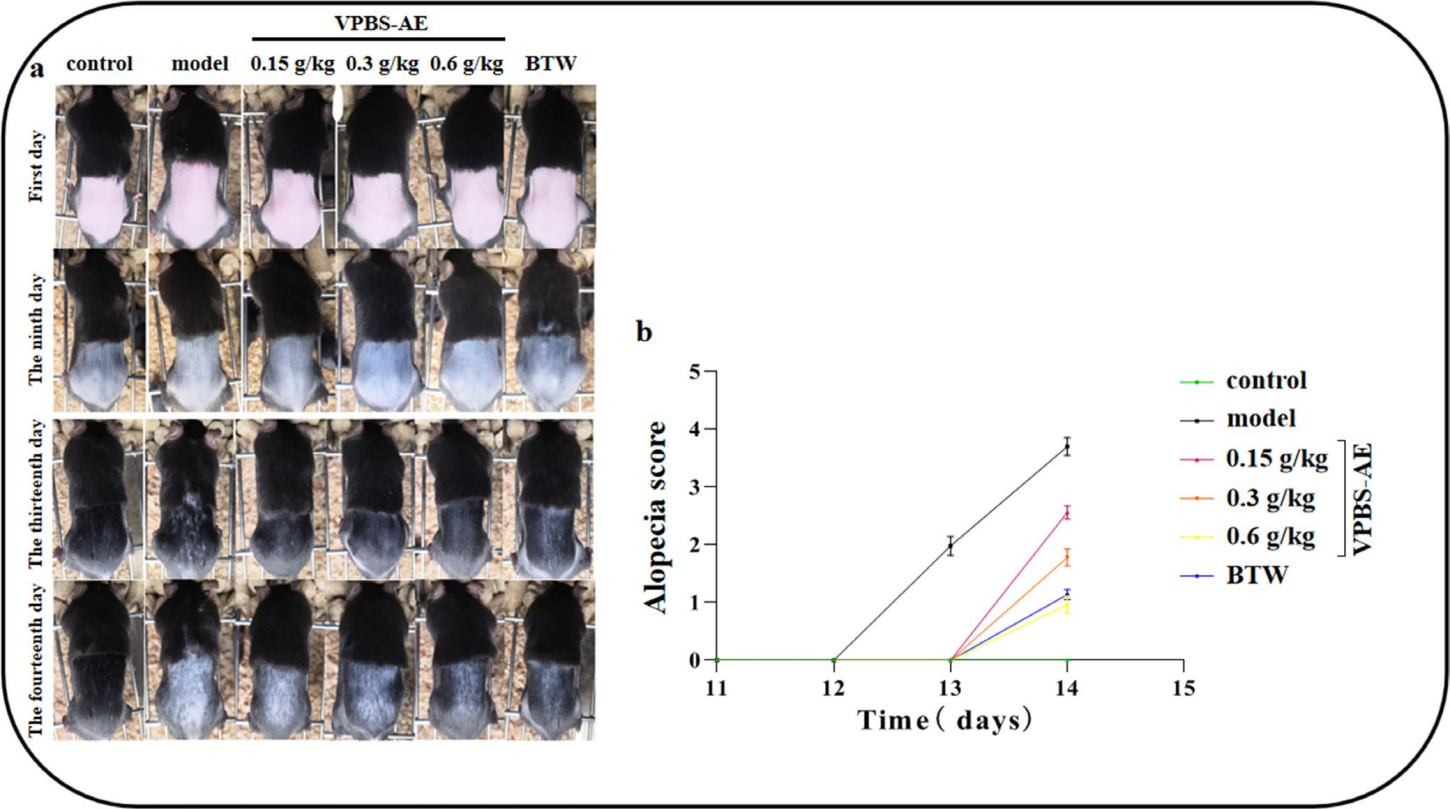
Effects of different doses of VPBS‐AE on cyclophosphamide‐induced alopecia in C57BL/6J mice. (a) Alopecia in C57BL/6J mice at different days. (b) Effects of VPBS‐AE on the alopecia score of C57BL/6J mice.

On Day 9, cyclophosphamide was injected intraperitoneally to establish a chemotherapy‐induced alopecia model. By Day 13, a substantial area of alopecia was observed in the model group, which was ameliorated following the administration of VPBS‐AE (Figure 7a). Meanwhile, the alopecia scores in VPBS‐treated groups significantly decreased compared to the model group (Figure 7b). These results demonstrate that VPBS‐AE can inhibit cyclophosphamide‐induced alopecia.

## 4 Discussion

**FIGURE 8:**
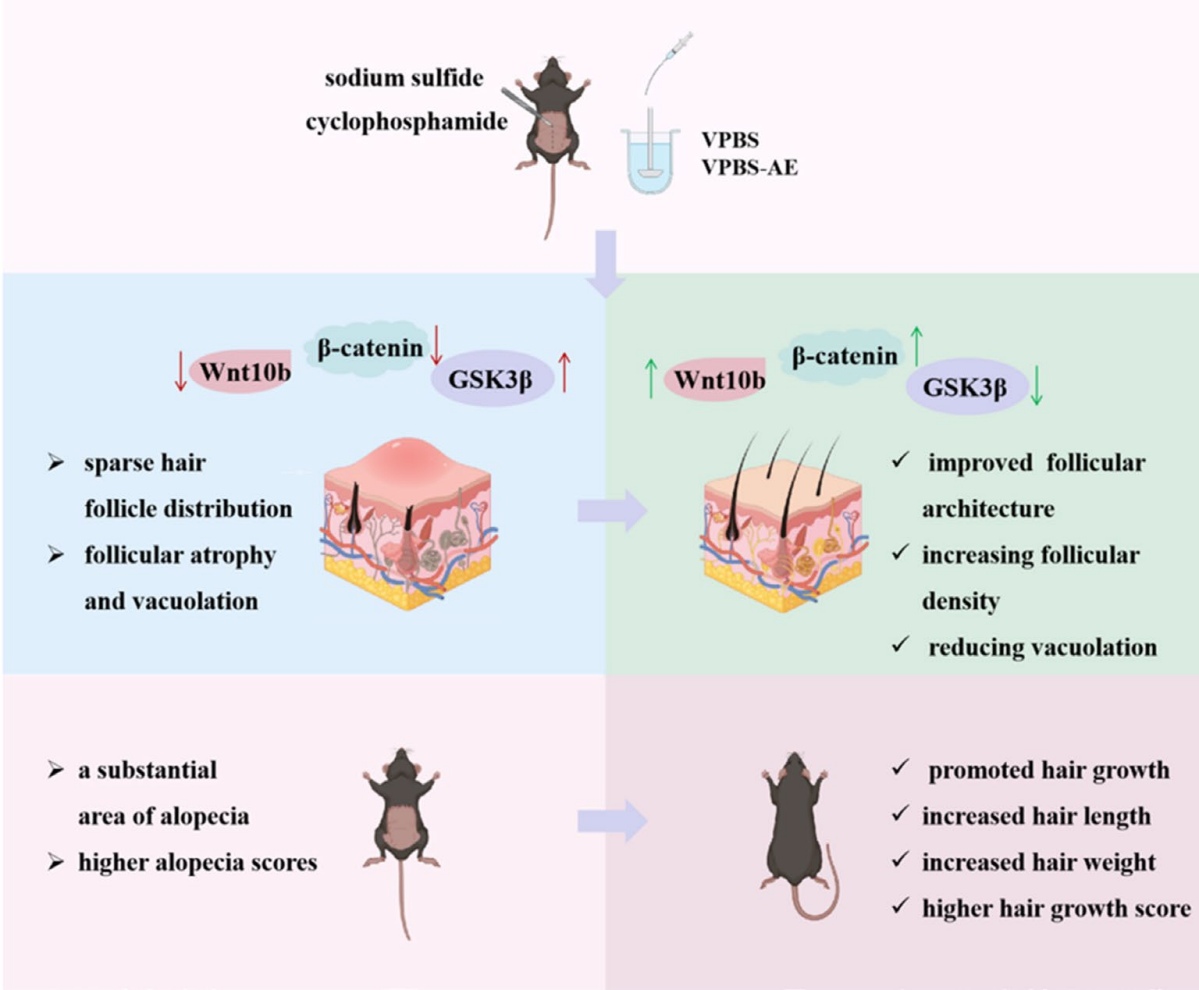
Effects and proposed mechanism of VPBS and VPBS‐AE on promoting hair growth and preventing alopecia.

This study demonstrated that VPBS and VPBS‐AE are capable of promoting hair growth and inhibiting alopecia in C57BL/6J mice. Specifically, a dosage of 3 g/kg VPBS and 0.3 g/kg VPBS‐AE exhibited the most pronounced effect. VPBS‐AE can diminish follicular vacuolization, augment dermal thickness, and alleviate follicular structural damage, thereby accelerating the hair‐growth process and mitigating hair loss induced by cyclophosphamide. These therapeutic effects may be mediated through activation of the Wnt/β‐catenin pathway (Figure 8).

Common experimental hair removal methods include chemical depilatory creams, sodium sulfide application, rosin/paraffin adhesion, and testosterone propionate induction. Depilatory creams exhibit heterogeneous compositions and depilatory efficacies across manufacturers. The rosin/paraffin method poses thermal injury risks due to high‐temperature application. Testosterone propionate is restricted to androgen‐dependent alopecia models, whereas sodium sulfide, a standardized chemical depilatory agent, provides consistent, nonthermal depilation (He et al. 2022). In this study, sodium sulfide achieved uniform depilation without residual follicular structures, yielding smooth murine skin. Although transient olfactory discomfort occurs, sodium sulfide induces minimal histopathological sequelae compared to alternative methods and enables rapid experimental model induction. These attributes established sodium sulfide as the optimal depilation strategy for this study.

To explore the hair‐promoting effect of different VPBS extractions, hair growth in mice was observed over a 14‐day period. For diseases related to alopecia, the growth of hair at the hair loss site is the most intuitive and favorable evaluation standard for drug efficacy. Hair length, hair weight, and hair growth score were analyzed as previous reports (Begum et al. 2023; Deng et al. 2021; Zhou et al. 2022). The results of hair growth observation clearly demonstrate that VPBS expedited the anagen of C57BL/6J mice and effectively promoted hair growth. In the aspect of hair length, following the administration of VPBS, a significant increase in the hair length of the mice was observed. When considering hair weight, after treatment with VPBS, the weight of the newly grown hair of the mice increased substantially. In terms of hair condition scoring, all scores after the administration of VPBS were higher than those of the model group. Through H&E staining experiments, the impact of VPBS‐AE on the number of hair follicles and dermal thickness in mice was investigated. The results demonstrate that VPBS‐AE can accelerate hair growth in mice, whereas the residue of VPBS‐AE fails to promote hair growth. This implies that the active hair‐promoting components are extracted into VPBS‐AE. Following treatment with VPBS‐AE, a significant increase in the hair length and weight of mice was observed, and the hair growth scores were higher than those of the model group. Histopathological analysis demonstrated that VPBS‐AE administration significantly increased follicular density, attenuated vacuolation, and enhanced dermal thickness in mice. These findings suggest that VPBS‐AE promotes hair regeneration through alleviating structural damage to the hair follicle.

The Wnt/β‐catenin pathway is closely associated with the cyclical growth of hair follicles. Wnt10b is expressed during the anagen of the hair follicle cycle, while it remains unexpressed during the catagen and telogen (Badria et al. 2020). GSK‐3β represents a pivotal signaling molecule within the Wnt/β‐catenin signaling pathway. During the anagen of the hair follicle, GSK‐3β undergoes phosphorylation and subsequent inactivation. This inactivation event results in an elevation of cytoplasmic free β‐catenin levels. The increased cytoplasmic free β‐catenin then translocates into the nucleus, where it activates downstream genes, such as c‐myc. This activation ultimately promotes the proliferation and differentiation of hair follicle cells (Iorizzo and Tosti 2018). Research has further demonstrated that GSK‐3β inhibitors are capable of inducing the nuclear transcription of β‐catenin. By regulating the cell cycle, these inhibitors play a role in promoting the development and growth of hair follicles (Chen et al. 2021). Moreover, β‐catenin is highly expressed during the anagen and lowly expressed during the telogen, suggesting a certain correlation between the expression of β‐catenin and the cyclic growth of hair follicles. Our findings revealed that sodium sulfide significantly suppressed Wnt10b and β‐catenin expression while elevating GSK3β levels in murine dorsal skin tissues compared to the control group. VPBS‐AE treatment restored Wnt10b and β‐catenin expression and suppressed GSK3β levels in a dose‐dependent manner. These results suggest that VPBS‐AE mitigates sodium sulfide‐induced follicular dysfunction by activating the Wnt/β‐catenin signaling pathway.

To assess antialopecia effects, a murine model was established via intraperitoneal cyclophosphamide injection (100 mg/kg) during telogen (Stage VI), 9 days postdepilation (Yoneda et al. 2021; Paus et al. 1994, 1996; Plonka et al. 2005; Chen et al. 2016; Lim et al. 2019). Macroscopic evaluation revealed extensive alopecia in the model group, confirming successful induction. VPBS‐AE treatment significantly reduced alopecic areas in a dose‐dependent manner (*p* < 0.05 vs. model group). Notably, alopecia onset occurred at 12 days postinduction in the model group versus 13 days in VPBS‐AE‐treated mice, indicating delayed hair loss progression and attenuated follicular structural damage. These findings suggest VPBS‐AE mitigates cyclophosphamide‐induced alopecia by prolonging anagen retention.

Black soybean is reported to contain bioactive compounds such as cyanidin 3‐*O*‐glucoside and procyanidins (Takahama et al. 2021). Takahashi et al. (1999) previously reported that procyanidins extracted from grape seeds exhibit growth‐promoting activity on murine hair epithelial cells in vitro. Additionally, these procyanidins stimulate anagen induction during hair cycle progression in vivo, thereby demonstrating the hair‐growing activity of procyanidins. Although the active components in VPBS that promote hair growth have not been clearly identified in this study, it can be speculated that procyanidins might be among the bioactive ingredients based on the literature reports. Moreover, further research on this aspect is essential in the future.

In summary, this study demonstrates that VPBS and VPBS‐AE exhibit significant potential as functional food candidates for promoting hair regeneration and mitigating chemotherapy‐induced alopecia. VPBS‐AE attenuated follicular damage, accelerated anagen entry, and preserved hair follicle integrity, likely via modulation of the Wnt/β‐catenin signaling pathway. However, several limitations warrant further investigation. For instance, while VPBS‐AE ameliorated cyclophosphamide‐induced hair loss, its efficacy against other etiologies of alopecia, such as androgenetic alopecia, autoimmune‐mediated hair loss, or stress‐induced telogen effluvium, remains unexplored. Additionally, mechanistic insights into bioactive constituents and bioavailability require rigorous validation. These studies will advance VPBS‐related products toward nutraceutical applications.

## Author Contributions

**Yue Zhang:** data curation (lead), formal analysis (lead), investigation (lead), methodology (lead), writing – original draft (equal). **Miaomiao Wu:** data curation (lead), investigation (lead), methodology (lead), writing – original draft (equal). **Jiasen Li:** data curation (supporting), formal analysis (supporting), investigation (supporting), methodology (supporting). **Chenghao Zhao:** investigation (supporting), resources (lead), writing – review and editing (supporting). **Tingyuan Zhou:** methodology (supporting), validation (lead), writing – review and editing (supporting). **Huiling Wei:** data curation (supporting), methodology (supporting). **Jiaxin He:** formal analysis (supporting), methodology (supporting). **Yao Yao:** conceptualization (equal), project administration (equal), supervision (equal), writing – review and editing (lead). **Juan Li:** conceptualization (equal), funding acquisition (lead), project administration (equal), supervision (equal), writing – review and editing (lead).

## Ethics Statement

All animal experiments were in compliance with the Regulations for the Administration of Experimental Animals in China, and approved by the Ethic Committee of Ningxia Medical University (Approval No. 2022‐Z030).

## Conflicts of Interest

The authors declare no conflicts of interest.

## Data Availability

The original contributions presented in the study are included in the article, further inquiries can be directed to the corresponding author.
